# Improving Blood Product Transfusion Premedication Plan Documentation: A Single-institution Quality Improvement Effort

**DOI:** 10.1097/pq9.0000000000000572

**Published:** 2022-06-14

**Authors:** Jitsuda Sitthi-amorn, Emily Denton, Erin Harper, Delia Carias, Saman Hashmi, Sakshi Bami, Allison Ast, Taylor Landry, Kenneth L. Pettit, Shilpa Gorantla, Anna Vinitsky, Yan Zheng, Liza-Marie Johnson

**Affiliations:** * Hospitalist Medicine Program, St. Jude Children’s Research Hospital, Memphis, Tenn.; † Department of Oncology, St. Jude Children’s Research Hospital, Memphis, Tenn.; ‡ Center of Advanced Practice, St. Jude Children’s Research Hospital, Memphis, Tenn.; § Department of Pharmacy and Pharmaceutical Sciences, St. Jude Children’s Research Hospital, Memphis, Tenn.; ¶ Department of Global Pediatrics, St. Jude Children’s Research Hospital, Memphis, Tenn.; ‖ Lewis Katz School of Medicine, Temple University, Philadelphia, Pa.; ** Office of Quality and Patient Care, St. Jude Children’s Research Hospital, Memphis, Tenn.; †† Department of Pathology, St. Jude Children’s Research Hospital, Memphis, Tenn.

## Abstract

**Introduction:**

Premedication with acetaminophen and/or diphenhydramine to prevent febrile nonhemolytic transfusion reactions and minor allergic transfusion reactions is a common practice based on historical recommendations. However, recent small randomized-controlled trials showed no benefit of premedication. This inconsistency leads to practice variability, which results in the inefficiency of our institution’s blood product ordering process. This project aimed to improve the number of transfusion encounters with premedication plan documentation from a baseline of 19% to 80% in 12 months.

**Methods:**

A multidisciplinary quality improvement (QI) team used QI tools to design interventions to improve the efficiency of the ordering process for blood products. Measures were tracked monthly and analyzed using statistical process control.

**Results:**

From September 2018 to January 2021, 5,351 blood product transfusion visits were scheduled. At baseline, 34% of patients received premedication, and 19% had premedication plans documented. Interventions included a passive computerized provider order entry alert, clinical care pathway development, and clinician education. Postimplementation, the average number of encounters with a premedication plan increased from 19% to 87%, whereas encounters receiving premedication decreased from 34% to 25%. There was no change in the average number of transfusion reactions (1.8 per 100 transfusions).

**Conclusions:**

Using QI methods, our team successfully standardized the blood product premedication plan documentation despite unclear best practices regarding blood product transfusion premedication. The team added premedication plan documentation training to new employee orientation for sustainability.

## INTRODUCTION

Febrile nonhemolytic transfusion reaction (FNHTR) and minor allergic transfusion reaction (MATR) are the two most common side effects of blood product transfusion.^1–5^ These reactions are often benign but can be time and resource-consuming, as they require evaluating the transfusion reaction and discarding blood products.^5,6^ Clinicians have been using acetaminophen and diphenhydramine to prevent FNHTR and MATR for several decades.^1,2,7–9^ However, FNHTR and MATR have become less frequent with the increasing use of leukoreduced products.^10–15^ The efficacy of premedication has not been extensively studied, perhaps due to the safety profile and low cost of acetaminophen and diphenhydramine and the benign nature of FNHTR and MATR. To date, there have been three small randomized-controlled trials on the efficacy of blood product premedication, and none showed the efficacy of premedication in reducing the incidence of FNHTR and MATR.^10,12,13^

There was a large variation in blood product premedication practice among clinicians at our institution.^11^Those who were against the practice cited the lack of efficacy. In contrast, supporters argued that the value of potentially preventing FNHTR and MATR outweighs both the cost and risk from acetaminophen and diphenhydramine and that the sample size of the trials was small.^8–10,12,13,16^ Nevertheless, this practice variation has led to the inefficiency of the blood product ordering process that negatively affected patient and clinician satisfaction at our institution. Providers had to spend time reviewing the electronic health record (EHR), looking for information about a patient’s premedication needs. Our team faced the challenges of addressing this practice variation when the best practice was unclear.

Standardization and availability of information can lead to process efficiency,^17,18^ and process efficiency is a component of patient and clinician satisfaction.^19–21^ Previous data showed that clinical pathway implementation, computerized provider order entry (CPOE), standardized documentation, education, and patient engagement could reduce variation and improve communication between healthcare teams.^9,16,22–25^ Thus, using a quality improvement (QI) approach, our team sought to improve ordering efficiency by increasing the rate of blood product premedication plan documentation. We aimed to improve the premedication plan documentation rate from a baseline of 19%–80% in 1 year.

## METHODS

### Context

Our institution is a free-standing pediatric academic medical center treating children with cancer and blood disorders. Patients are seen in different primary clinics based on diagnosis (ie, hematology, neuro-oncology). In addition, the hospital runs a weekend clinic for both scheduled and unscheduled visits, and the clinic sees patients from all primary clinics. The weekend clinic is staffed by a dedicated team of hospitalist-based physicians, advanced practice providers, and nurses who are not affiliated with a disease-specific primary clinic. Many patients are scheduled in the weekend clinic to assess blood product transfusion needs.

At the beginning of the project, the blood product premedication ordering process was inefficient and confusing. There was a specific section in the hospital EHR to document the blood product premedication plan, but it was not routinely filled out. As a result, it was unclear which patient(s) needed blood product premedication and if recommendations for premedication should be documented by primary oncologists, cross-covering oncologists, or transfusion medicine physicians. In addition, before administering blood products, nurses often heard from a child’s parent that the child needed a premedication and had to ask for orders from physicians or advanced practice providers. This need caused a delay in patient care and led to patient and staff dissatisfaction.

### Interventions

**Fig. 1.**
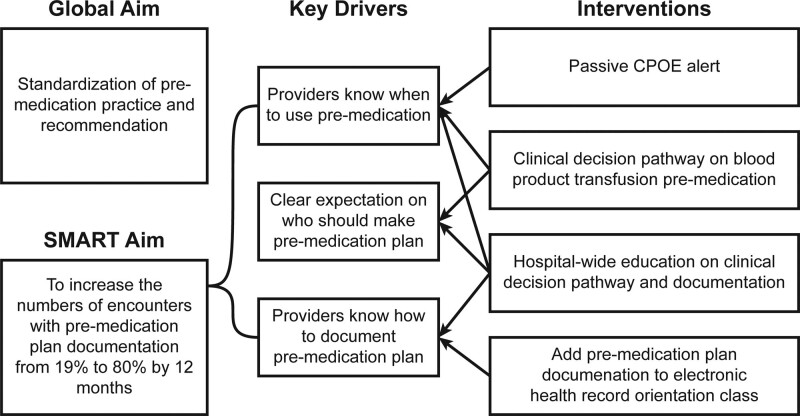
Key drivers for improved premedication plan documentation rate.

A multidisciplinary QI team comprising pediatric oncologists, transfusion medicine physicians, advanced practice providers, nurses, clinical pharmacists, and QI project managers used the QI methodology as per the Institute of Healthcare Improvement model^26^ to address the issue. Key drivers to improve premedication plan documentation included clear recommendations on when to use premedication, who should determine the premedication plan, and how to document the premedication plan (Fig. 1). Change concepts for the interventions included agreeing on expectations, standardization, giving people access to information, using reminders, and training.^26^

To set clear recommendations on what premedication to use and who should determine the premedication plan, the QI team sought institutional expert opinion, performed a literature review,^3,4,10–13^ and created an institutional clinical decision pathway to prevent FNHTR and MATR, defining the indication for premedication (**see figure S1, Supplemental Digital Content 1,** which describes clinical care pathway for blood product transfusion premediation, http://links.lww.com/PQ9/A380). It also clearly stated that any cross-covering physicians or advanced practice providers who evaluate patients for the transfusion reaction should be making the clinical decision on future premedication needs (**see figure S2, Supplemental Digital Content 1,** which describes process map of tasks and responsibilities of different team members during transfusion reaction evaluation, http://links.lww.com/PQ9/A380). The institutional Pharmacy and Therapeutics Committee, which includes members from all clinical areas, reviewed the clinical decision pathway and approved the implementation.

Since the recommendations in the clinical pathway are based on common practice and expert opinion, the QI team did not audit provider adherence. Instead, the team focused on the rate of premedication plan documentation, as this could potentially improve the efficacy of the blood product ordering process. First, the team collaborated with the Department of Information Technology and created a passive CPOE reminder linking premedication plan documentation with all transfusion orders. In addition, team members conducted institution-wide education on the clinical decision pathway and premedication plan documentation. The team focused on education efforts for advanced practice providers working in primary clinics as they do most clinical documentation. The QI team tailored the education to each primary clinic to meet their needs.

### Study of Interventions

To better understand the rationale behind the institutional premedication practice, the QI team reviewed the rationales listed in the premedication plan documentation of all patients who received premedication for blood product transfusion. The information was used to design additional interventions to standardize blood product premedication practice at our institution.

### Measures

Process measure was the monthly average of premedication plan documentation rate in patients receiving premedication for transfusion. The outcome measure was the monthly average premedication usage rate in relation to the total number of blood product transfusion encounters. For this project, we defined ambulatory transfusion encounters where acetaminophen and/or diphenhydramine were given within 30 minutes of check-in as a transfusion with premedication. The balancing measure was the average percentage of transfusion encounters with transfusion reaction evaluation performed. Details on all measures, including their operational definition and source of data, are described in Table 1.

**Table 1.** Operational Definition and Sources of Data for of Measures

Measure	Type of Measure	Operational Definition		
		Numerator	Denominator	Sources of Data
Premedication plan documentation rate	Process	Encounters with premedication plan documentation	Transfusion encounters that patients received premedication for blood product transfusion, defined as receiving acetaminophen or diphenhydramine within 30 min after check-in time	Manual review (numerator) Electronic database (denominator)
Premedication rate	Outcome	Transfusion encounters that patients received premedication for blood product transfusion, defined as receiving acetaminophen or diphenhydramine within 30 min after check-in time	All scheduled blood product transfusion encounters	Electronic database
Transfusion reaction evaluation rate	Balancing	All transfusion encounters that required transfusion reaction evaluation	All scheduled blood product transfusion encounters	Electronic database

### Analysis

The QI team tracked all measures on control charts monthly to understand the process in real time. We used data from September 2018 to August 2019 to calculate baseline performance. Centerline shift was based on pre-established rules for special cause variation if there were additional interventions. The rules used for this project were eight points on the same side of centerlines and a point beyond the control limits.^26^ In addition, we performed a funnel plot^27^ of the premedication plan documentation rate for the five primary clinics that sent patients to the weekend clinic to better understand each clinic’s unique baseline practice. Control charts were created with QI-Charts 2.0 excel extension (Process Improvement Products, Austin, Tex., www.pipproducts.com).

This project received the Non-Human Research Determination (QI) from the local Institutional Review Board.

## RESULTS

**Fig. 2.**
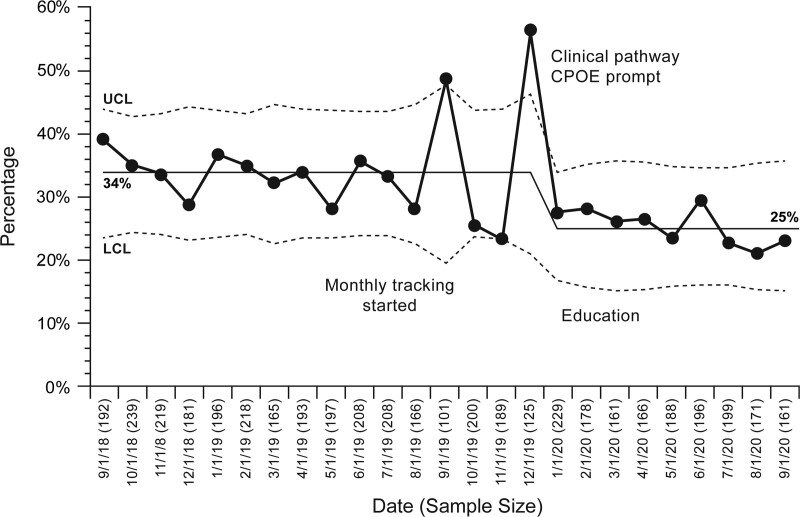
Transfusion encounters with premedication given. LCL, lower control limit; UCL, upper control limit.

**Fig. 3.**
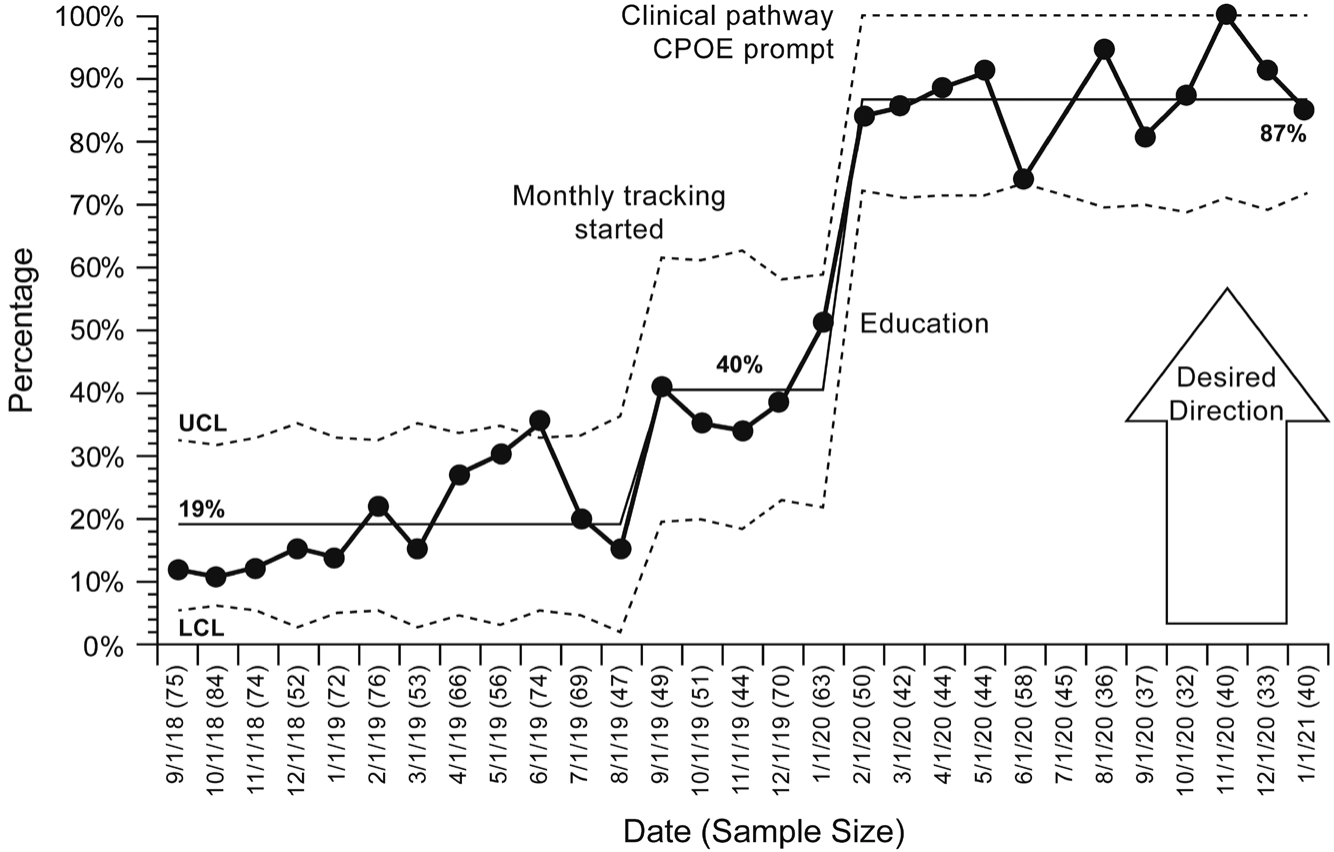
Premedication plan documentation rate. LCL, lower control limit; UCL, upper control limit.

From September 2018 to January 2021, there were 5,351 scheduled blood product transfusion visits (an average of 185 visits per month). Patients received leukoreduced and irradiated packed red blood cells or single-donor apheresis platelets. At baseline, an average of 34% of patients received premedication for blood product transfusion (Fig. 2). For encounters where patients received premedication, 19% had the premedication plan documented (Fig. 3). An average of 1.8% of all transfusion encounters was evaluated for a possible transfusion reaction (**see figure S3, Supplemental Digital Content 1,** which describes transfusion reaction evaluation rate during the length of the project, http://links.lww.com/PQ9/A380).

The project leader started planning and forming the team in May 2019. From May 2019 to January 2020, the team drafted the clinical care pathway and presented it for the institution’s Pharmacy and Therapeutics Committee to review. The pathway was approved for implementation in January 2020. Although we did not implement any formal intervention, the rate of premedication plan documentation has increased to 40% based on the preparation work alone (Fig. 3).

**Fig. 4.**
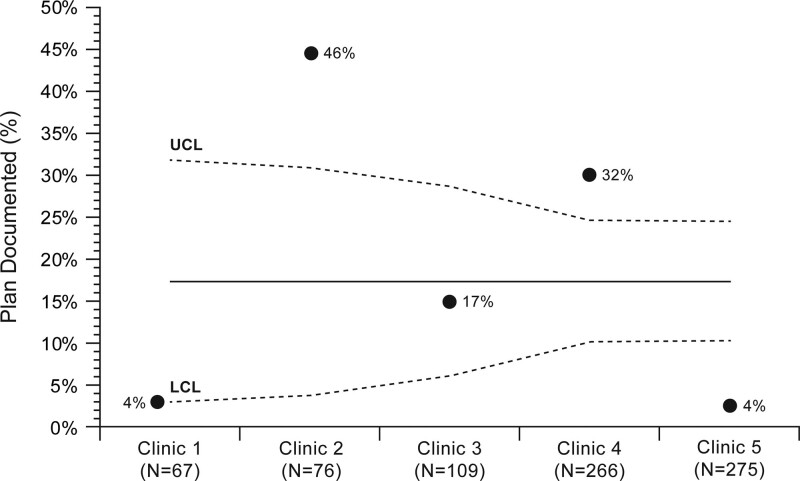
Funnel plot of baseline premedication plan documentation by the clinic. LCL, lower control limit; UCL, upper control limit.

In February 2020, the team implemented the CPOE prompt and listed the clinical care pathway in the hospital’s online formulary. There was no specific training module on blood product premedication plan documentation for advanced practice providers who performed most of the clinical documentation. The funnel plot showed that the premedication plan documentation rates varied among different primary clinics (Fig. 4). This information helped the QI team design an education plan specific to each area. For example, clinic 5 had the lowest baseline premedication plan documentation rate because most clinicians were not aware of the functionality of the premedication plan. Therefore, the education was focused on entering the documentation in the EHR. In clinic 4, clinicians knew how to document the premedication plan but did not know what to recommend. Therefore, we focused the education on the new clinical care pathway.

The QI team partnered with the Center for Advanced Practice Providers to introduce the clinical care pathway and premedication plan documentation during educational sessions in February 2020. The QI team showed the funnel plot to the providers to gain buy-in. As a result, from Spring 2020 onward, the average premedication plan documentation rate increased to 87% and remained at about 80% for 12 months (Fig. 3). In addition, the blood product transfusion premedication usage decreased from 34% to 25%, despite not conducting clinical care pathway adherence auditing (Fig. 2). The transfusion reaction rate remained stable at 1.8% (**see figure S3, Supplemental Digital Content 1,** http://links.lww.com/PQ9/A380).

There were 114 documented rationales for transfusion premedication listed in the EHR. These rationales included history of MATR (N = 38, 33%), history of severe allergic reaction (N = 29, 25%), history of febrile reaction (N = 16; 14%), prevention of FNHTR for patients with chemotherapy-induced neutropenia (N = 20, 18%), preference of patients or family members (N = 10, 9%), and others (N = 1, 1%) (see **figure S4, Supplemental Digital Content 1,** which describes rationales for blood product transfusion premedication, http://links.lww.com/PQ9/A380).

## DISCUSSION

Practice variation in premedication for transfusion of blood products caused process inefficiency and negatively impacted patient and staff satisfaction at our institution. However, using multiple QI tools, our team successfully improved documentation of the blood product premedication plan and reduced the usage of premedication. Our study shows that the standardization of clinical practice is possible even when the best practice is unclear.

As noted in previously published studies, our QI team successfully standardized local clinical practice by implementing a triad of passive CPOE alerts,^22,25^ clinical care pathways,^9,23^ and clinical education.^24^ The rate of premedication plan documentation improved during the planning stage without formal intervention, suggesting that providers changed practice when they realized they were being monitored.^28^ Although the QI team did not focus on enforcing adherence to the clinical care pathway, the hospital’s blood product premedication rate decreased after pathway implementation. This observation supports our theory that guidelines and clinical pathways can reduce variation in healthcare.^23,24^ The stable rate for transfusion reactions despite decreased premedication usage is consistent with several studies showing that acetaminophen and diphenhydramine were ineffective in preventing FNHTR and MARs.^10–14^

Interestingly, many providers ordered acetaminophen to prevent febrile neutropenia in patients who receive blood product support while experiencing chemotherapy-induced neutropenia. However, to our knowledge, the risks and benefits of acetaminophen in preventing FNHTR in this group of patients have not been explored. This insight is useful for our effort to further standardize the blood product premedication practice at our institution and others treating a large pediatric oncology population.

This study has several limitations. First, the process measure was the number of encounters with premedication plan documentation, not the number of unique patients. Since some patients receive multiple transfusions in the same month, the rate of premedication plan documentation may be biased by multiple patients receiving repeated transfusions. Furthermore, we defined patients who received premedication based on acetaminophen and/or diphenhydramine within 30 minutes after checking in for the blood product transfusion encounter. However, we could not determine if the patients received both medications for premedication purposes. Thus, the true incidence of premedication remains unknown.

Moreover, the transfusion reaction rate was captured through the number of transfusion reaction evaluations ordered. This number may be different than the actual transfusion reaction rate. However, both measures were the best proxy measures that the QI team could access without completing a manual medical record review. Although not 100% accurate, these numbers were representative of the process and outcome of interests. Using them allowed the QI team to execute the project with available resources. In addition, the team did not specifically measure the time spent during the blood product ordering and administration process. This fact limited the ability to demonstrate the impact of the intervention on the clinic flow. Finally, the QI team did not focus on ensuring adherence to the clinical care pathway. This decision was intentional since the best practice for blood product transfusion premedication is unclear. Nonetheless, the team reduced the number of blood product premedication rates at our institution.

Our analysis showed that clinical care standardization is possible even when the best clinical practice is unclear.^7,8^ For sustainability, the QI team incorporated the blood product premedication plan education into EHR training for new fellows and advanced practice providers. In addition, our interventions may be useful for other teams working on clinical care standardization, especially in situations with limiting evidence for clinical practice. Moving forward, we plan to study the role of acetaminophen in preventing FNHTR in patients with chemotherapy-induced neutropenia. The knowledge will help us refine the clinical care pathway for blood product transfusion premedication.

## ACKNOWLEDGMENTS

The authors thank Vani Shanker, PhD, ELS, for editing the manuscript.

The authors presented the preliminary data at the American Society of Pediatric Hematology-Oncology Annual Conference (April 2021, virtual event).

The institution is supported by the ALSAC. Funding for the work done by T.L. was supported by the National Cancer Institute grant R25CA23944.

## DISCLOSURE

J.S. led and completed the project while working at St Jude Children’s Research Hospital and is currently an employee of Janssen Research and Development, Wayne, PA. The other authors have no financial interest to declare in relation to the content of this article.

## Supplementary Material


