# Supplementary material for: Improving Blood Product Transfusion Premedication Plan Documentation: A Single-institution Quality Improvement Effort
Source: Pediatr Qual Saf. 2022 Jun 14;7(3):e572. doi: 10.1097/pq9.0000000000000572 (PMC9197348; doi:10.1097/pq9.0000000000000572)
Supplement: Supplementary file 1 [file pqs-7-e572-s001.pdf]

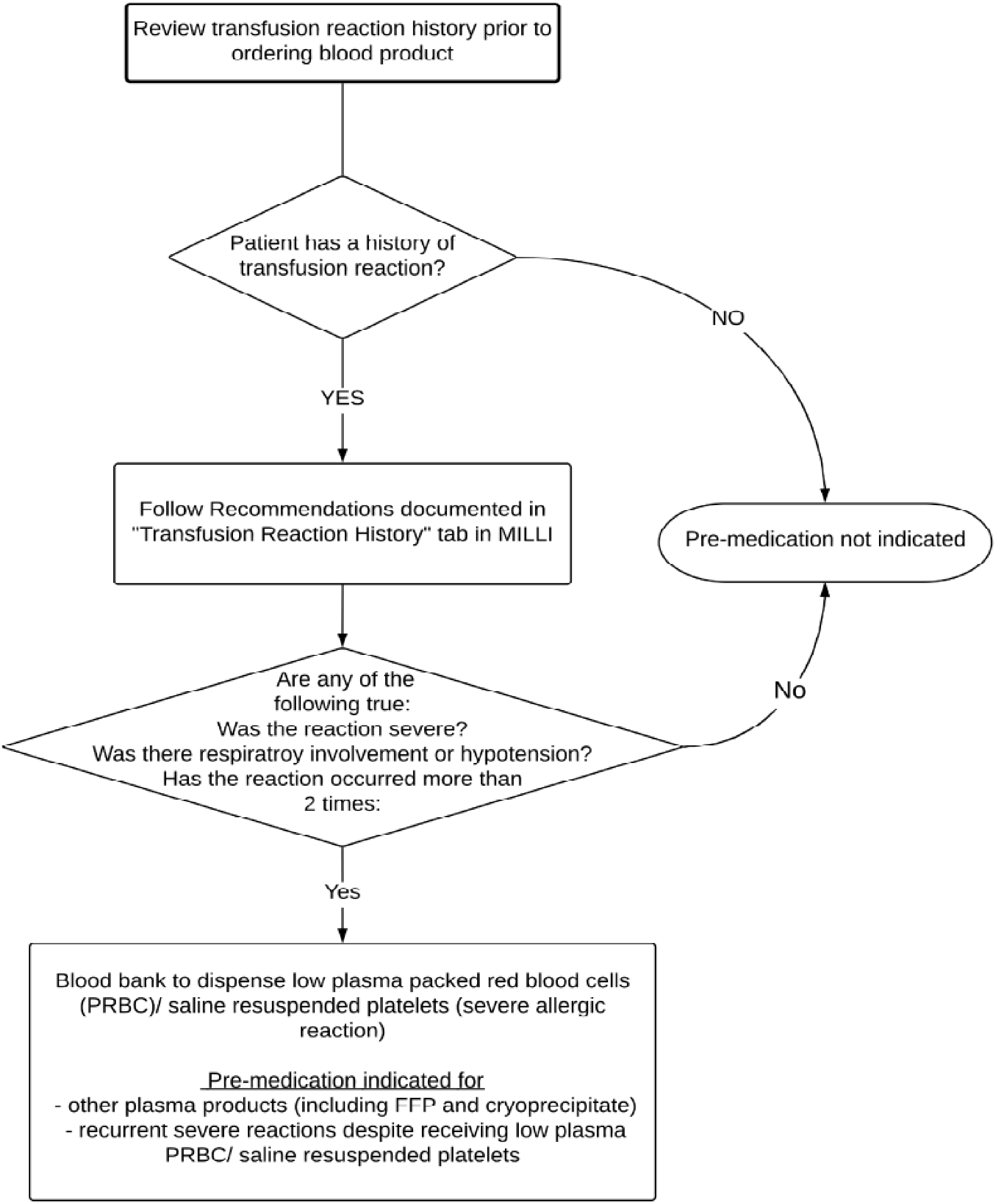
Review transfusion reaction history prior to ordering blood product
Patient has a history of transfusion reaction?
NO
YES
Follow Recommendations documented in "Transfusion Reaction History" tab in MILLI
Pre-medication not indicated
Are any of the following true:
Was the reaction severe?
Was there respiratroy involvement or hypotension?
Has the reaction occurred more than 2 times:
No
Yes
Blood bank to dispense low plasma packed red blood cells (PRBC)/ saline resuspended platelets (severe allergic reaction)
Pre-medication indicated for
- other plasma products (including FFP and cryoprecipitate)
- recurrent severe reactions despite receiving low plasma PRBC/ saline resuspended platelets

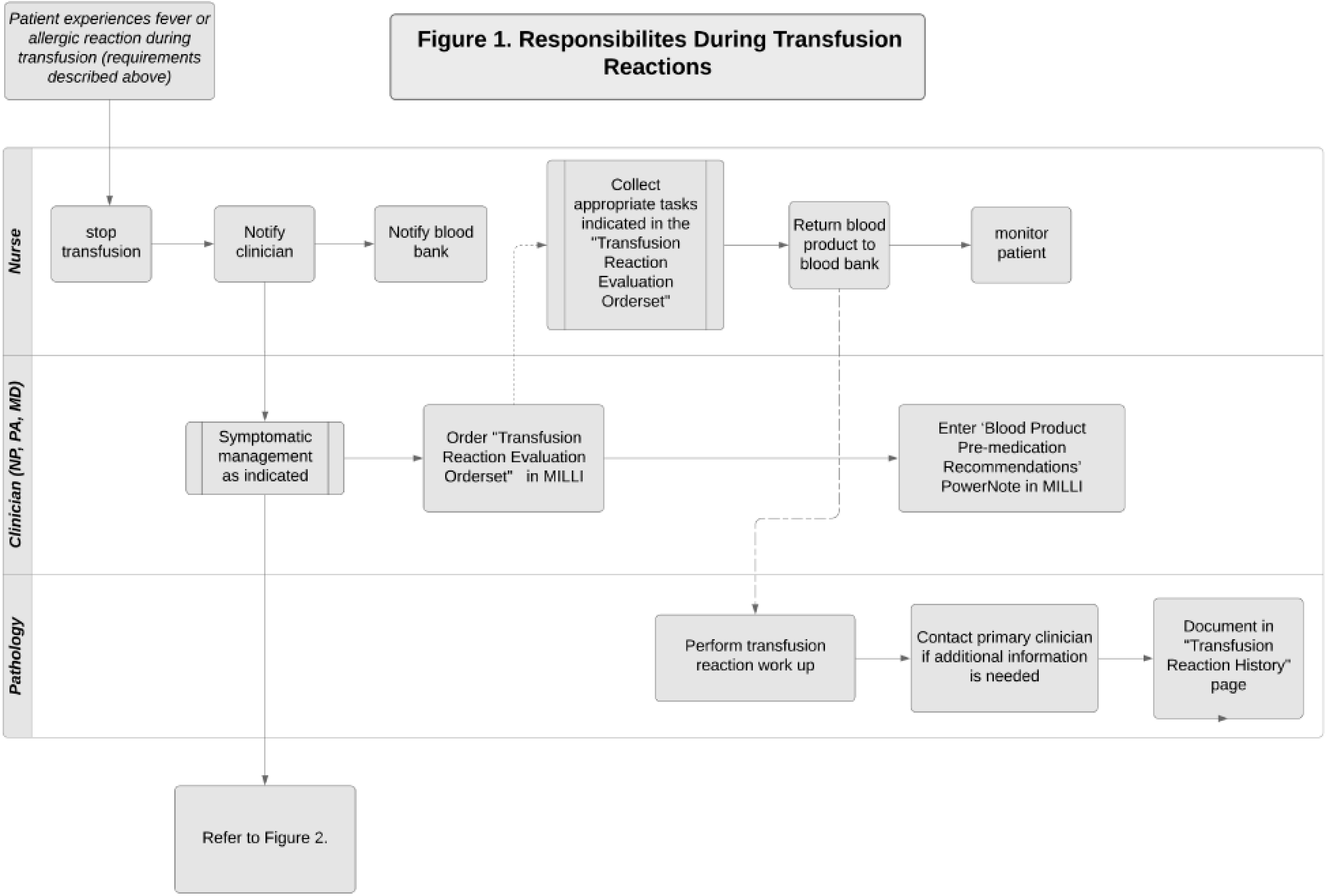

Figure 1. Responsibilites During Transfusion Reactions

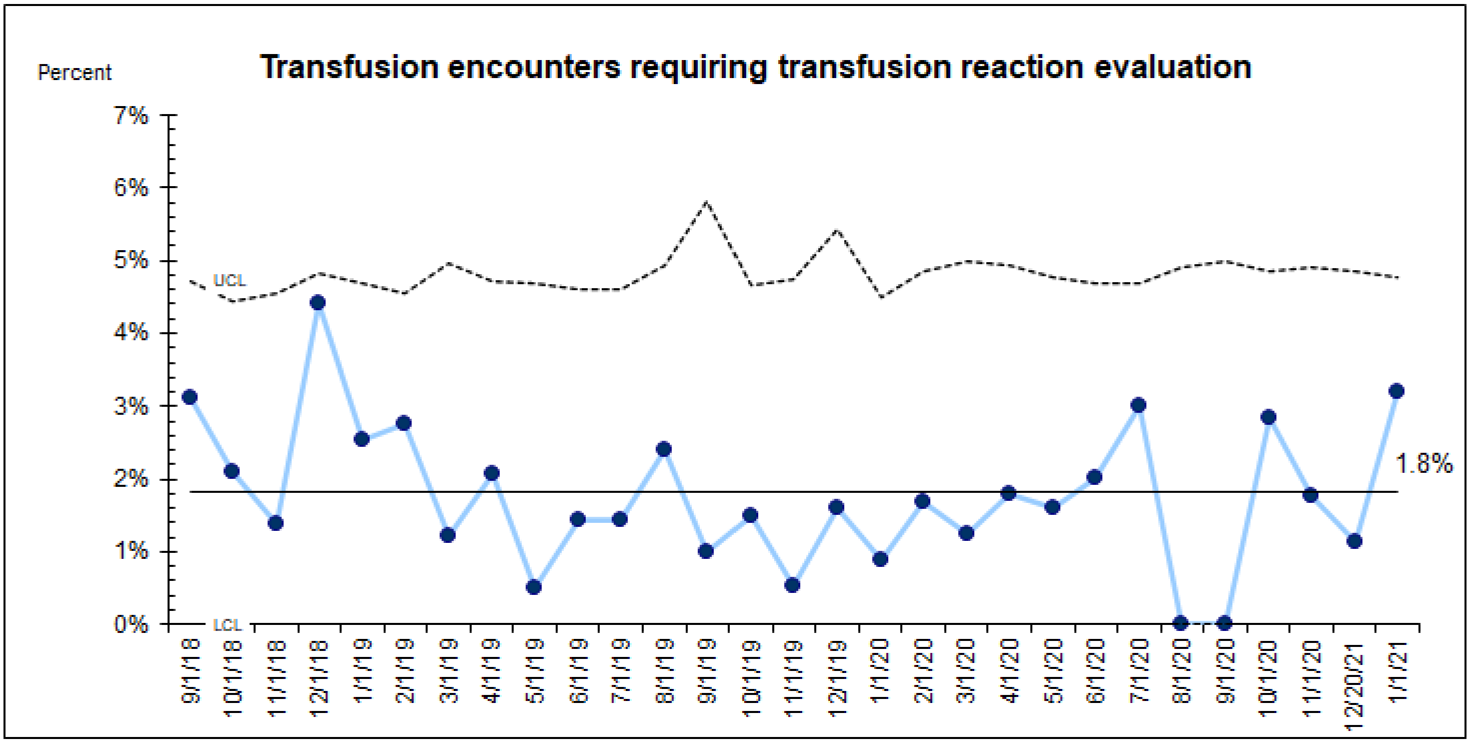
Transfusion encounters requiring transfusion reaction evaluation
Percent
7%
6%
5%
4%
3%
2%
1%
0%
UCL
LCL
1.8%
9/1/18
10/1/18
11/1/18
12/1/18
1/1/19
2/1/19
3/1/19
4/1/19
5/1/19
6/1/19
7/1/19
8/1/19
9/1/19
10/1/19
11/1/19
12/1/19
1/1/20
2/1/20
3/1/20
4/1/20
5/1/20
6/1/20
7/1/20
8/1/20
9/1/20
10/1/20
11/1/20
12/20/21
1/1/21

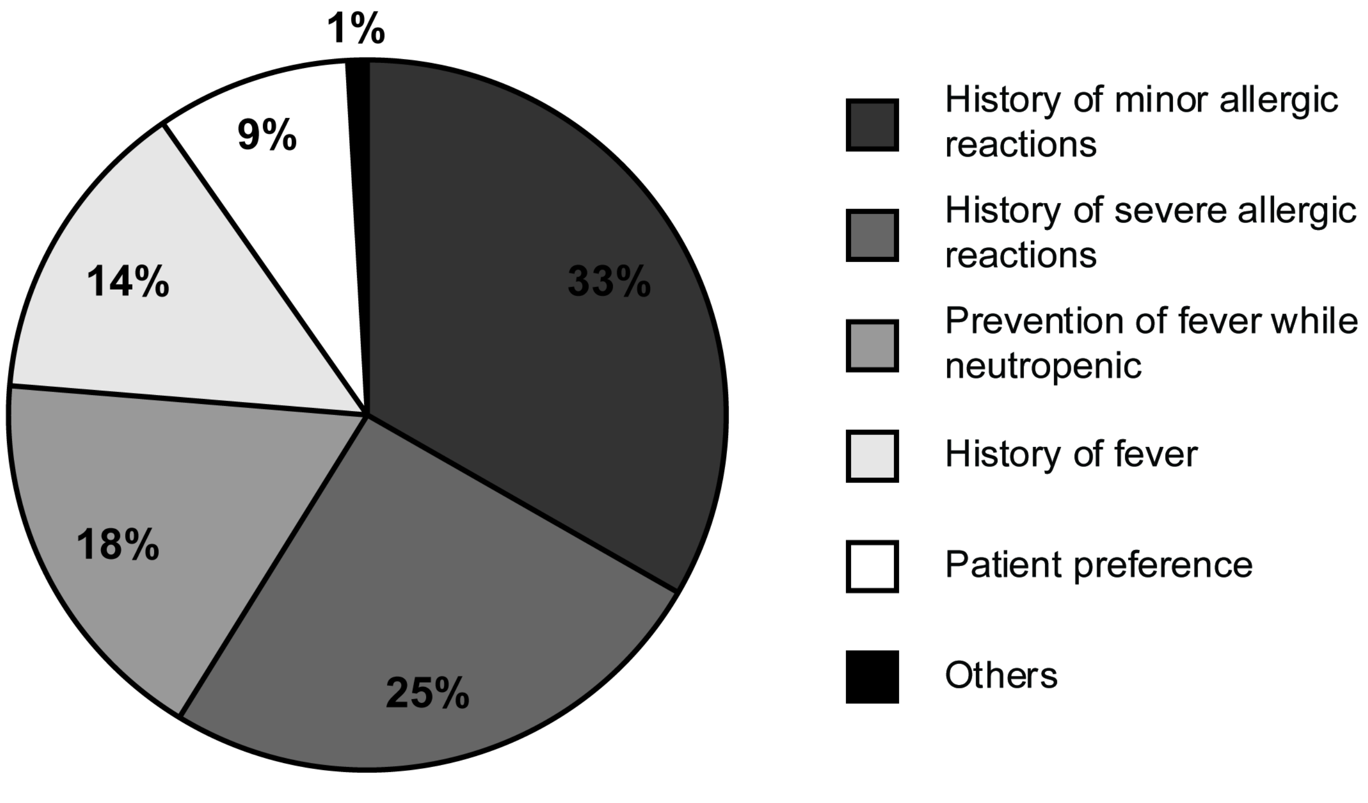
1%
9%
14%
33%
18%
25%
History of minor allergic reactions
History of severe allergic reactions
Prevention of fever while neutropenic
History of fever
Patient preference
Others

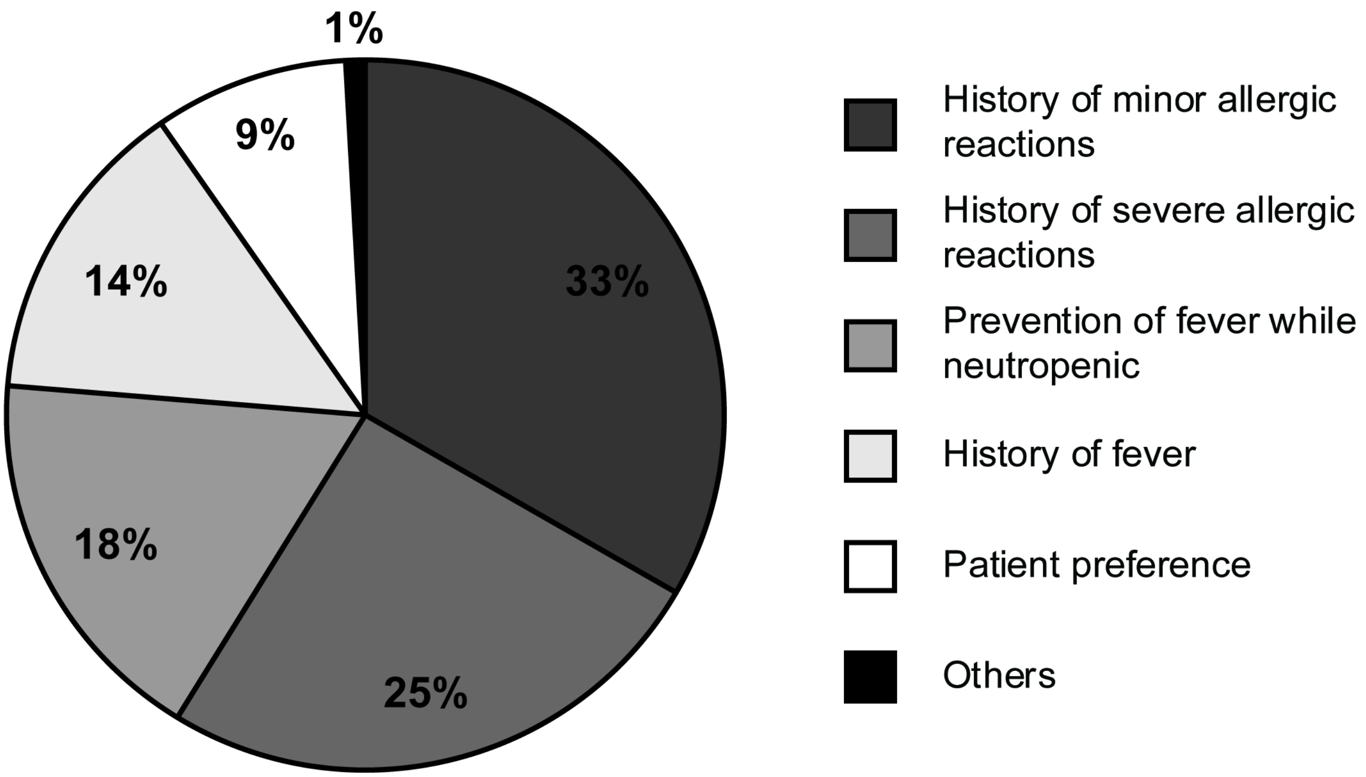
1%
9%
14%
33%
18%
25%
History of minor allergic reactions
History of severe allergic reactions
Prevention of fever while neutropenic
History of fever
Patient preference
Others
